# Micro- and Macrovascular Complications in Diabetes Mellitus: Preclinical and Clinical Studies

**DOI:** 10.1155/2019/2161085

**Published:** 2019-02-17

**Authors:** Érika B. Rangel, Cláudia O. Rodrigues, João R. de Sá

**Affiliations:** ^1^Hospital Israelita Albert Einstein, São Paulo, SP, Brazil; ^2^Department of Medicine, Nephrology Division, Federal University of São Paulo, SP, Brazil; ^3^Interdisciplinary Stem Cell Institute, University of Miami Miller School of Medicine, Miami, FL, USA; ^4^Department of Molecular and Cellular Pharmacology, University of Miami Miller School of Medicine, Miami, FL, USA; ^5^Department of Medicine, Division of Endocrinology, Federal University of São Paulo, SP, Brazil

Diabetes mellitus (DM) is a worldwide public health problem that affects millions of people from all age, gender, and racial and ethnic groups. DM is the leading cause of blindness and amputation and contributes substantially to kidney disease, cardiomyopathy, and cerebrovascular and peripheral artery diseases. Of importance, recent advances in biology and medicine have introduced new technologies to study the molecular pathology underlying DM-related complications, the development of novel strategies to treat these conditions, and the evaluation of outcomes.

The present special issue has been designed to stimulate the continuing efforts to develop novel drugs and therapeutic targets and new perspectives of combined therapies for decreasing micro- and macrovascular complications in DM. It includes one review article and ten original research papers, from leading and emerging scientists with diverse expertise and interests, and covers three thematic areas: (a) epidemiology and pathogenesis of DM-related complications; (b) microvascular complications (nephropathy, retinopathy, and neuropathy); and (c) macrovascular complications (cardiovascular disease, stroke, and peripheral artery disease). In all these thematic areas, pathophysiological and molecular mechanisms are discussed and novel drug-target therapies, as well as stem cell-based therapy, are documented in either preclinical or clinical studies. Of importance, future preclinical studies and smaller clinical trials are warranted before proceeding to pivotal trials.

In the paper of the present special issue entitled “Prevalence of Chronic Complications, Their Risk Factors, and the Cardiovascular Risk Factors among Patients with Type 2 Diabetes Attending the Diabetic Clinic at a Tertiary Care Hospital in Sri Lanka,” M. H. Arambewela et al. reported the prevalence of micro- and macrovascular complications of 3,000 patients with type 2 DM (T2DM) and their risk factors in a single center. To note, the study comprised predominantly female patients (~75%), which are usually underscored in epidemiologic studies. Their main findings included that increased age, disease duration, and glycated hemoglobin (HbA_1c_) were the main risk factors for microvascular disease and diabetic foot, while age was the only risk factor for macrovascular complications. In addition, occurrence of coronary artery disease (CAD), peripheral neuropathy, diabetic foot, and lower extremity amputation was significantly higher among male individuals when compared to female individuals. Collectively, gender differences may be taken into account when providing healthcare management and allocate resources for prevention and treatment of DM-related complications [1].

Besides traditional risk factors for macrovascular DM-related complications, emerging data of single nucleotide polymorphisms (SNPs) provide new insights for the risk of hyperglycemia and the development of these complications and their response to current treatment. In the study of X. Ma et al. entitled “Polymorphisms in the Glucagon-Like Peptide 1 Receptor (*GLP-1R*) Gene Are Associated with the Risk of Coronary Artery Disease in Chinese Han Patients with Type 2 Diabetes Mellitus: A Case-Control Study,” eleven haplotype-tagging SNPs for GLP-1R were tested. They found that patients with the GG genotype at rs4714210 had a lower CAD risk when compared to patients with other genotypes, even when other known CAD risk factors were evaluated. GLP-1R agonists are associated with a decrease in well-known risk factors for heart disease, such as HbA_1c_, fasting blood glucose, body weight, waist circumference, systolic and diastolic blood pressures, total cholesterol, and triglycerides [2]. Moreover, understanding GLP-1R polymorphism may help predict individual response to CAD treatment, although randomized controlled and multicenter trials are further required to verify the importance of GLP-1R polymorphism.

In the Evaluation of Lixisenatide in Acute Coronary Syndrome (ELIXA) multicenter randomized and placebo-controlled trial, 6,068 patients with T2DM who had a recent coronary event were enrolled into this trial and followed for a median of 25 months [3]. The addition of lixisenatide, a GLP-1 agonist, to standard care did not significantly alter the rate of major cardiovascular (CV) events or other serious adverse events, including renal and vascular disease occurrence. Further exploratory analysis of the ELIXA trial documented that lixisenatide reduced the progression of urinary albumin-to-creatinine ratio in macroalbuminuric patients and was associated with a lower risk of new-onset macroalbuminuria after adjustment for HbA_1c_ and other traditional renal risk factors [4]. In the study entitled “Retinopathy, Neuropathy, and Subsequent Cardiovascular Events in Patients with Type 2 Diabetes and Acute Coronary Syndrome in the ELIXA: The Importance of Disease Duration” of J. P. Seferovic et al., both retinopathy and neuropathy were associated with a primary composite outcome (CV death, nonfatal MI (myocardial infarction), stroke, or hospitalization for unstable angina); CV composite (CV death, nonfatal MI, stroke, and hospitalization for heart failure (HF)); myocardial infarction; HF hospitalization; and all-cause mortality. However, when retinopathy and neuropathy were adjusted to T2DM duration, no association was found, and only T2DM duration was related to those outcomes. These findings point out the relevance of adjusting DM duration to other variables when clinical trials are critically analyzed, as well as when novel therapeutic strategies are tested in DM setting.

Stem cell-based therapy is considered a promising strategy for the treatment of DM and DM-related complications [5]. In the review of this special issue entitled “Addressing Stem Cell Therapeutic Approaches in Pathobiology of Diabetes and Its Complications”, B.-Y. Peng et al. summarized the main findings of stem cell-based therapy, with a focus on multipotent stem cells (hematopoietic stem cells and mesenchymal stem cells (MSCs) from different sources) and pluripotent stem cells (embryonic stem cells and inducible pluripotent stem cells) in micro- and macrovascular DM-related complications in both T1DM and T2DM settings. Pathophysiological and molecular mechanisms in DM, such as the increase in oxidative stress and advanced glycation end products (AGEs) accumulation and the decrease in endothelial progenitor cells function, were addressed. The broad spectrum of stem cell pleiotropic properties was also discussed, including paracrine effects (immunomodulation, secretion of growth, and antiapoptotic and antifibrotic factors) and direct differentiation into damaged tissue. Of importance, efficacy of stem cell-based therapy requires further studies addressing the homing capacity, the most important source of stem cells and their route of injection, the number of infusions, and the severity of tissue damage and endpoints, which ultimately will reach definite conclusions about the therapeutic potential of stem cells and their impact on clinical outcomes [6].

As documented in several clinical trials, experimental findings on stem cell-based therapy for human disease are controversial [7]. MSC-based therapy reduced the severity of acute allograft kidney rejection and opportunistic infection after kidney transplant [8], although not affecting allograft survival and kidney function. Similarly, a single infusion of allogeneic MSCs stabilized or improved estimated glomerular filtration ratio in patients with moderate to severe DN [9]. However, no benefits of MSCs infusion were noted in patients with acute kidney injury after cardiac surgery [10]. In the study of K. W. Lee et al. entitled “Renal Ischemia-Reperfusion Injury in a Diabetic Monkey Model and Therapeutic Testing of Human Bone Marrow-Derived Mesenchymal Stem Cells,” a cynomolgus monkey model of streptozotocin-induced DN combined to acute renal ischemia-reperfusion injury, MSC treatment promoted amelioration of functional parameters and attenuated morphological kidney damage. The results obtained from this study will contribute to set the basis for establishing further investigation on the therapeutic potential of MSCs for treatment of kidney disease in other preclinical and clinical studies.

Endothelial dysfunction (ED) plays also a critical role in DM-related complications and represents an imbalance in the production of vasodilator factors, which results in prothrombotic and atherogenic effects in the vasculature [11]. Furthermore, ED in DM setting is associated with vasoconstriction, leukocyte adherence, platelet activation, mitogenesis, prooxidation, impaired coagulation, and decreased nitric oxide production. CKD is a potential contributor to the pathogenesis of ED [12]. M. N. Coutinho et al., in their study entitled “There Is No Impact of Diabetes on the Endothelial Function of Chronic Kidney Disease Patients” highlighted endothelial dysfunction in T2DM patients with CKD and compared their findings to nondiabetic patients with CKD. In DM-CKD patients, they observed more frequently obesity, previous MI, myocardial revascularization, and a higher number of endothelial progenitor cells and pulse wave velocity when compared to non-DM-CKD patients. Surprisingly, uremic toxins were the main determinants of ED in DM-CKD patients and not DM per se. These findings unravel important aspects of endothelial progenitor cell modulation in DM-CKD setting and have important biological implications for therapeutic application of stem cells therapy in that population. However, future studies are urgently needed to fully gain mechanistic insights when uremic toxins and hyperglycemia are both present.

Taking a step forward in our understanding of DN pathogenesis, C. Wang et al. evaluated in their study entitled “Artificially Cultivated *Ophiocordyceps sinensis* Alleviates Diabetic Nephropathy and Its Podocyte Injury via Inhibiting P2X7R Expression and NLRP3 Inflammasome Activation” the therapeutic potential of *Ophiocordyceps sinensis* (ACOS), which corresponds to a fungus-caterpillar complex formed after the fungus infects the larva of the moth. ACOS has been used for centuries in China and Asian countries. Mechanistically, ACOS reduced the expression of the P2X7 receptor (P2X7R) and NLRP3 inflammasome (NLRP3, ASC, and caspase 1) and downstream effectors (IL-1*β* and IL-18), yet decreasing podocyte injury *in vitro* and *in vivo* in a rat model of DN (low dose of streptozotocin and high-fat diet). P2X7 receptors are highly expressed on macrophages and are essential components of proinflammatory signaling in multiple tissues. In diabetic patients, renal P2X7R expression is associated with severe mesangial expansion, impaired glomerular filtration ratio, and increased interstitial fibrosis. These findings were similarly found in P2X7R-deficient mice [13]. Correspondingly, P2X7R activation enhanced the release of MCP-1 in human mesangial cells cultured under high-glucose conditions. Therefore, inhibition of P2X7R may represent a novel target in DN setting. The NOD-like receptor family pyrin domain containing 3 (NLRP3) inflammasome is a newly recognized and potent inflammatory mediator that induces inflammatory responses in several disorders, including DN. There is an interconnectivity between NLRP3 inflammasome, inflammation, and oxidative stress, which are activated by damage-associated molecular patterns (DAMPs, e.g., hyperlipidemia, hyperglycemia, receptor for AGES, islet amyloid polypeptide protein, and oxidized low-density protein) and ultimately drive micro- and macrovascular DM-related complications [14]. A key aspect of NLRP3 inflammasome activity in DN nephropathy is its modulation by antidiabetic drugs used in routine clinical practice, such as insulin, biguanides, sodium-glucose cotransporter-2 (SGLT2) inhibitors, sulfonylureas, thiazolidinediones, and dipeptidyl peptidase-4 (DPP-4) inhibitors [15].

One of these drugs, the SGLT2 inhibitors (empagliflozin, dapagliflozin, and canagliflozin), led to significantly lower rates of death from cardiovascular causes (38%), hospitalization for heart failure (35%), and death from any cause (32%) in T2DM patients, as documented in the randomized controlled trial EMPA-REG Outcomes (*n* = 7,020 individuals) with a 3.1-year follow-up [16]. Besides its effect on macrovascular DM-related complications, empagliflozin was associated significantly with lower rates of incident or worsening of DN (12.7% vs. 18.8%). Doubling of the serum creatinine and renal replacement therapy initiation also decreased by 44% and 55%, respectively [17]. In the study of E. H. Cho et al. entitled “Potent Oral Hypoglycemic Agents for Microvascular Complication: Sodium-Glucose Cotransporter 2 Inhibitors for Diabetic Retinopathy,” they studied 49 individuals with T2DM under SGLT2 inhibitors (empagliflozin or dapagliflozin) treatment. They found that these drugs decreased the risk of diabetic retinopathy (DR) progression, even when adjusted for age, duration of DM, initial DR grade, and HbA_1c_ levels. These findings identified gaps that need to be addressed in further studies, yet full spectrum of SGLT expression and its role in the eye is poorly understood [18].

The pathogenesis of DR includes several hyperglycemia-mediated mechanisms, e.g., protein C kinase and polyol pathway activation, AGE accumulation, and increase in hexosamine pathway flux. These mechanisms promote changes in retinal blood flow, increase in vascular permeability, ED, altered growth factor signaling, thickening of capillary basement membrane, pericyte loss, microaneurysms, hemorrhages, apoptosis, and increase of reactive oxygen species [18]. One of these features, the thickening of capillary basement membrane is associated with deposition of extracellular matrix components, such as fibronectin, collagen IV, and laminin, which lead ultimately to microvascular occlusion and retinal hypoperfusion. In the study of G. Song et al. entitled “Effects of High Glucose on the Expression of LAMA1 and Biological Behavior of Choroid Retinal Endothelial Cells,” laminin alpha-1 (LAMA1) expression was investigated in retinal choroidal vascular endothelial cells (RF/6A line). Surprisingly, authors found an inverse correlation of LAMA1 expression and glucose concentration, as well as a lower capacity of proliferation, migration, and adhesion of retinal choroidal vascular endothelial cells. These findings indicate that LAMA1 may exert a protective effect against DR, although future *in vitro* and *in vivo* studies are needed to verify these findings. Moreover, recreating the microenvironment of the eye will provide mechanistic insights of LAMA1 role in DR setting.

Peripheral artery disease (PAD) is particularly debilitating as it may result in acute and chronic inferior limb lesions and amputations in DM setting. In addition, PAD is extremely costly for the patient and financially impacts the economy as a range of prophylactic actions and drug-related and endovascular/surgical treatments may be essential. Diabetic foot affects 15% of diabetic patients, yet neuropathy, neuroischemia, and infections have an interconnectivity in determining the worsening of the lesions [19]. Moreover, almost 85% of all amputations are preceded by a foot ulceration that deteriorates to a severe gangrene or infection. In the paper entitled “Distribution of Microbes and Drug Susceptibility in Patients with Diabetic Foot Infections in Southwest China,” M. Wu et al. evaluated the microbial distribution through collection of deep ulcer secretion and drug susceptibility among diabetic foot ulcers (DFUs), using Wagner classification, in 428 hospitalized patients. They documented a distinct distribution and type of bacteria in accordance to Wagner classification (grades 3-5 had mainly gram-negative bacilli) and duration of DFUs (chronic ulcer was also associated with gram-negative bacilli in 54.2%). Of importance, knowledge of the microbial etiology in DFUs and understanding antibiotic resistance is critical for an effective management and treatment of these infected lesions. Likewise, conventional diagnostic methods combined to molecular techniques for bacterial identification and quantification may represent a powerful approach in DFU setting.

A promising therapy for diabetic foot is the bacteria-killing nanotechnology Bio-Kil socks, as addressed in the paper of D. Lu et al. and entitled “Insoles Treated with Bacteria-Killing Nanotechnology Bio-Kil Reduce Bacterial Burden in Diabetic Patients and Healthy Controls”. Their findings showed that Bio-Kil socks efficiently reduced bacterial growth in both diabetic patients and healthy individuals, mainly in Gram-positive bacteria, which has important implications for the design of future studies. Recent advances in the field point out to the development of insoles with arch design and ulcer isolations for effective stress reduction in a diabetic foot, and these novel insoles are designed with a skin-like material [20]. To note, these findings may positively impact the outcomes of diabetic patients with DFUs.

Collectively, the present special issue provides new findings in micro- and macrovascular diabetic complications and future management of these complications. Of importance, further knowledge of the pathophysiological and molecular mechanisms involved in the onset and progression of DM-related complications is needed for not only to treat these complications but also to curtail their progression, which ultimately may provide a better care to patients.
